# Infectious Diseases

**Published:** 1893-06

**Authors:** 


### INFECTIOUS DISEASES.

The nature of the infectious diseases, chief of which are Asiatic cholera, yellow fever, smallpox, diphtheria, scarlet fever, measles, typhus fever, typhoid fever, and others, are not generally understood by those who so much dread them. While cholera and yellow fever, when they break out in an epidemic, are the most virulent and deadly for a time, they do not on the average cause as many deaths as diphtheria, which stands at the head of infectious diseases for its fatality.

This disease is especially fatal to children, and is one of the most difficult to prevent from spreading. This disease is carried around easily in clothes, food and air, and its spread is very difficult to stop. It will even spread from corpses, and from burial places where the bodies have not been properly interred.

Next to this disease, typhoid fever stands as the most fatal, and as a rule half as many die from this annually as from diphtheria. Typhoid is not so infectious, but it is very deadly in its attack. It is communicated more through food and drinking water. Scarlet fever stands pretty close to typhoid fever in its fatality, and their relative death rate is about four to five. In this disease, as well as diphtheria, typhus fever and smallpox, funerals in a church should never be permitted, as this will frequently spread the disease rapidly. The whooping cough comes next in its deadliness, followed closely by measles. Smallpox is really a very rare disease, owing to prevention by vaccination. Its relative death rate is extremely small compared with the other diseases.

Of late, consumption has been placed among infectious diseases, and the death rate from this disease is one and a half times greater than from diphtheria, more than three times as great as typhoid, over four times as great as scarlet fever, ten times as great as whooping cough, thirteen times as great as measles, and thirty-five times as great as smallpox, or nearly equal to all of the other diseases combined.

